# Impact of dietary supplementation of glycocalyx precursors on vascular function in type 2 diabetes

**DOI:** 10.1152/japplphysiol.00651.2024

**Published:** 2024-10-31

**Authors:** James A. Smith, Francisco I. Ramirez-Perez, Katherine Burr, Juan D. Gonzalez-Vallejo, Mariana Morales-Quinones, Neil J. McMillan, Larissa Ferreira-Santos, Neekun Sharma, Christopher A. Foote, Luis A. Martinez-Lemus, Jaume Padilla, Camila Manrique-Acevedo

**Affiliations:** ^1^Department of Nutrition and Exercise Physiology, University of Missouri, Columbia, Missouri, United States; ^2^NextGen Precision Health, University of Missouri, Columbia, Missouri, United States; ^3^Division of Endocrinology and Metabolism, Department of Medicine, University of Missouri, Columbia, Missouri, United States; ^4^Department of Medical Pharmacology and Physiology, University of Missouri, Columbia, Missouri, United States; ^5^Center for Precision Medicine, Department of Medicine, University of Missouri, Columbia, Missouri, United States; ^6^Harry S. Truman Memorial Veterans’ Hospital, Columbia, Missouri, United States

**Keywords:** diabetes, dietary supplement, endothelial function, glycocalyx

## Abstract

Degradation of the endothelial glycocalyx in type 2 diabetes (T2D) is thought to contribute to impaired shear stress mechanotransduction, leading to endothelial dysfunction and the development of cardiovascular disease. Herein, we tested the hypothesis that restoration of the endothelial glycocalyx with dietary supplementation of glycocalyx precursors (DSGPs, containing glucosamine sulfate, fucoidan, superoxide dismutase, and high-molecular weight hyaluronan) improves endothelial function and other indices of vascular function in T2D. First, in db/db mice, we showed that treatment with DSGP (100 mg/kg/day) for 4 wk restored endothelial glycocalyx length, as assessed via atomic force microscopy in aortic explants. Restoration of the glycocalyx with DSGP was accompanied by improved flow-mediated dilation (FMD) and reduced arterial stiffness in isolated mesenteric arteries. Further corroborating these findings, the treatment of cultured endothelial cells with that same mixture of glycocalyx precursors promoted glycocalyx growth. Next, as an initial step to investigate the translatability of these findings, we conducted a pilot (*n* = 22) double-blinded randomized placebo-controlled clinical trial to assess the effects of DSGP (3,712.5 mg/day) for 8 wk on endothelial glycocalyx integrity and indices of vascular function, including FMD, in Veterans with T2D. Contrary to the hypothesis, DSGP neither enhanced endothelial glycocalyx integrity nor improved vascular function indices relative to placebo. Together, these findings conceptually support the notion that restoration of the endothelial glycocalyx can lead to improvements in vascular function in a mouse model of T2D; however, DSGP as a therapeutic strategy to enhance vascular function in individuals with T2D does not appear to be efficacious.

**NEW & NOTEWORTHY** Endothelial glycocalyx degradation in type 2 diabetes (T2D) is thought to contribute to impaired shear stress mechanotransduction, leading to vascular dysfunction. The findings of this study support the notion that restoration of the endothelial glycocalyx using a dietary supplementation of glycocalyx precursors can lead to improvements in vascular function in diabetic mice. However, the utilized dietary supplement as a therapeutic strategy to enhance vascular function in individuals with T2D is not efficacious.

## Graphical abstract

**Figure:**
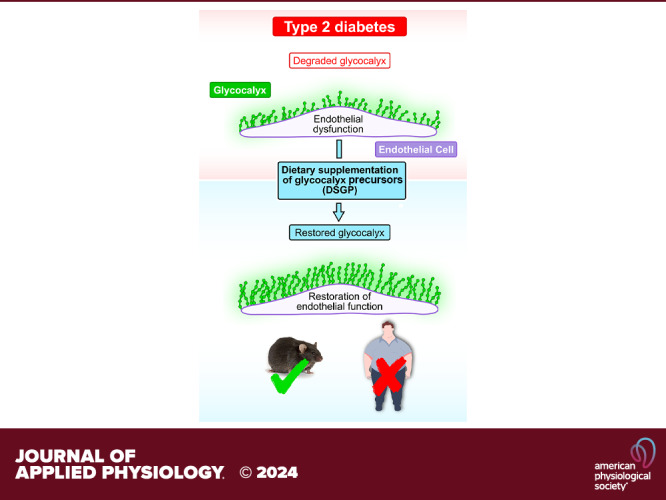

## INTRODUCTION

The prevalence of type 2 diabetes (T2D) continues to increase in the United States and worldwide (1–3). In particular, T2D is widespread among the Veteran population, and the Veterans Affairs (VA) administration spends $1.5 billion annually on diabetes care (4, 5). Importantly, T2D contributes to the staggering rates of cardiovascular disease (CVD) and cardiovascular mortality in this population (6). Indeed, it is estimated that eight out of 10 patients with T2D will die from CVD (7). Endothelial dysfunction, a central vascular feature of T2D, plays a vital role in the pathogenesis of CVD (8–11). Thus, a better understanding of the mechanisms underlying endothelial dysfunction in T2D can be exploited for therapeutic gain in preventing and treating CVD.

The endothelial glycocalyx, a negatively charged layer of membrane-bound glycoproteins, proteoglycans, and glycosaminoglycans located on the surface of vascular endothelial cells, plays an essential role in transmitting mechanical forces (i.e., shear stress) into the cell (12, 13). This process, known as mechanotransduction, is critical for maintaining endothelial health, such that if impaired, it leads to dysfunction of the endothelium. Indeed, degradation of these luminal mechanosensing structures, which occurs in T2D owing to oxidative stress, inflammation, hyperglycemia, disturbed or reduced flow profiles, among other mechanisms (12, 14–19), contributes to impaired shear stress mechanotransduction and, consequently, endothelial dysfunction (17, 20, 21). Therefore, therapeutic strategies aimed to restore the endothelial glycocalyx should be considered to combat vascular complications in T2D.

To that end, we report that restoration of the endothelial glycocalyx with dietary supplementation of glycocalyx precursors (DSGPs, containing glucosamine sulfate, fucoidan, superoxide dismutase, and high-molecular weight hyaluronan) increases glycocalyx length, improves flow-mediated dilation (FMD), indicative of enhanced shear stress mechanotransduction, and lessens arterial stiffness in a mouse model of T2D (i.e., db/db mice). Further supporting these findings, we show that treating cultured endothelial cells with this cocktail of glycocalyx precursors promotes glycocalyx formation. Based on these observations, we proceeded to conduct a double-blinded, randomized, placebo-controlled clinical trial to examine the effects of 8 wk of DSGP on endothelial glycocalyx integrity and indices of vascular function, including FMD, in Veterans with T2D. We hypothesized that, compared with placebo, DSGP treatment would enhance the integrity of the endothelial glycocalyx and improve overall vascular function in Veterans with T2D.

## METHODS

### Animal Studies

All animal procedures were approved by the Animal Use and Care Committee at the University of Missouri and performed in accordance with National Institutes of Health guidelines. Twelve-week-old db/db female mice were obtained from The Jackson Laboratory. Mice were allocated to two treatment groups: dietary supplementation of glycocalyx precursors (DSGPs; Endocalyx) 100 mg/kg/day or vehicle (i.e., peanut butter; 6.7 kcal/g of food, JIF, The J.M. Smucker Company, Orrville, OH) for 4 wk. Peanut butter, palatable to rodents, was chosen to keep the supplement in suspension and ensure intake, which was monitored daily throughout the intervention. The peanut butter was administered independently of the chow. A cohort of age-matched db/+ mice was used as a reference control. Standard chow for feeding (5053-PicoLab Rodent Diet 20, LabDiet) was provided ad libitum, and mice had unlimited access to water. Two mice were housed in each cage under 12-h light and dark cycles. Caretakers monitored the health of the mice daily. For euthanasia, isoflurane inhalation (2%, AKORN Animal Health) with room air (250 mL/min) was used to induce surgical-plane anesthesia, followed by pneumothorax and exsanguination. Arteries were removed for functional and mechanical assessments. Each mouse was considered an experimental unit for analyzing all study outcomes.

#### Ex vivo aortic vasomotor function.

Aortic 2-mm rings were prepared by removing perivascular fat and connective tissue and then were mounted in wire myography organ bath chambers (620M, Danish Myo Technology, Hinnerup, Denmark) containing warmed physiological saline solution gassed with 95% O_2_–5% CO_2_ and kept at 37°C, as previously described (22). To assess viability, arterial rings were constricted with KCl (80 mM). Subsequently, aortas were pre-constricted with the prostaglandin H2/thromboxane A2 receptor agonist, U-46619 (20 nM). Cumulative increasing concentrations of acetylcholine (ACh, 10^−9^ to 10^−5^ M) and the nitric oxide (NO)-donor sodium nitroprusside (SNP, 10^−9^ to 10^−4^ M) were added to the vessel bath to determine arterial relaxation responses. ACh was used to assess endothelium-dependent relaxation, whereas SNP was used to assess endothelium-independent relaxation.

#### Ex vivo arterial vasomotor and mechanical responses.

Mesenteric arteries were collected to assess vascular functional and mechanical responses via pressure myography, as previously described (22, 23). The isolated arteries were cannulated and mounted onto pressure myographs set at an intraluminal pressure of 70 mmHg and exposed to a series of sequential 10^−5^ M phenylephrine preconstrictions followed by increasing concentrations of ACh (10^−9^ to 10^−5^ M) or SNP (10^−8^ to 10^−4^ M) in half-log increments every 2 min to assess endothelium-dependent and -independent vasodilatory responses, respectively. Following this, the cannulated vessels were exposed to a calcium-free buffer containing 2 mM ethylene glycol-bis(β-aminoethyl ether)-*N*,*N*,*N*′,*N*′-tetraacetic acid, and 0.1 mM adenosine to achieve passive conditions. Intraluminal pressures were then varied from 5 to 120 mmHg at intervals of 2 min, with internal diameter and wall thickness recorded to assess vascular remodeling and stiffness, as described previously (22, 23). For detailed equation descriptions and parameters implemented to assess mechanical and biophysical properties of arteries, see the work by Wenceslau et al. (24). Each artery originated from one mouse for outcome assessments.

#### Ex vivo endothelial stiffness.

The elastic modulus (stiffness) of the aortic endothelial surface was measured in explants of thoracic aorta from mice using atomic force microscopy (AFM, MFP-3D AFM 89 Asylum Research Inc., Goleta, CA), as described previously (22, 25). Briefly, a segment of the thoracic aorta was isolated, opened longitudinally, and secured onto a glass slide with Cell-Tak adhesive. The samples were maintained at room temperature (∼25°C) and subjected to repeated cycles of nanoindentation and retraction. A custom Python script analyzed the generated indentation curves to calculate the force curves and determine the elastic modulus of the endothelial surface using the Hertz model (26).

#### Ex vivo glycocalyx integrity.

Similar to the method used earlier to measure the elastic modulus, endothelial glycocalyx length and stiffness in aortic explants were assessed via AFM nanoindentation experiments using a 5.0 µm spherical probe attached to a cantilever (NovaScan), with a spring constant of 0.02 N/m. To perform the curve analysis needed to determine glycocalyx length and stiffness, a custom code was implemented following the recommendations outlined by Targosz-Korecka et al. (20). For each 2 mm^2^ aortic explant, at least 50 nanoindentation curves were obtained from 7 randomly chosen locations within the sample. The curves were recorded during application of a maximal loading force of 1.0 nN at a speed of 0.25 µm/s. Curves that deviated from the established mathematical model (i.e., unbiased) were discarded.

### Endothelial Cell Culture Experiments

Human umbilical vein endothelial cells (HUVECs, CC-2519, Lonza) were cultured in complete VascuLife EnGS medium (LL-0002, Lifeline Cell Technologies) supplemented with EGM medium (CC-3124, Lonza) and 2% fetal bovine serum. HUVECs (5 × 10^4^ cells/well; passaged 5/6) were seeded onto 96-well plates and cultured at 37°C under 5% CO_2_ for 24 h. Then, 100 mU/mL neuraminidase (N2876, Sigma), with or without a cocktail of glycocalyx precursors in reduced fetal bovine serum (0.5%) cell media, was used to treat cells for 48 h. We have previously shown that this concentration of neuraminidase is an effective approach to cause glycocalyx destruction (26). To prepare the cocktail of glycocalyx precursors, a mortar and pestle were used to grind the content of the capsules to a fine powder, and 0.2 mg/mL was solvent extracted in 95% EtOH. The soluble extract was diluted 1:4,000 into cell media. After treatments, cells were fixed in 4% paraformaldehyde (15710, Electron Microscopy Sciences) prior to immunofluorescence staining and imaging. To evaluate the extent of glycocalyx coverage on endothelial cells, fixed cells were washed twice with phosphate-buffered saline and incubated for 30 min with 3 mg/mL Alexa-488-conjugated wheat-germ agglutinin (WGA-Alexa-555, W32464, Molecular Probes). Nuclei were stained with 4′,6-diamidino-2-phenylindole (DAPI, No. D9542, Sigma-Aldrich, 1:500 dilution). A microplate reader (BioTeK Synergy H1, Agilent, Santa Clara, CA) equipped with area scanning mode was used to collect fluorescence measurements. Excitation/emission wavelengths were set at 539/580 nm for WGA and 377/477 nm for DAPI. The WGA signal was normalized to DAPI fluorescence to control for variations in cell number per well.

### Clinical Trial

All human study procedures conformed to the Declaration of Helsinki and were approved by the University of Missouri Institutional Review Board (Protocol 2062542), the R&D committee at the Harry S Truman VA, and the Med/Surg VA Data Monitoring Committee. The double-blinded, randomized, placebo-controlled clinical trial was registered at ClinicalTrials.gov (NCT05205005) with an FDA IND 164629. Written informed consent was obtained from all subjects before any study procedures. Men (*n* = 23) and women (*n* = 1) Veterans aged 45 to 74 yr with a diagnosis of T2D, overweight or obesity (BMI 25–45 kg/m^2^), an HbA1c <9%, and fasting blood glucose <200 mg/dL at screening visit were recruited from the Harry S Truman VA Hospital. Participants who, within the past year, had experienced a cardiovascular event or had uncontrolled hypertension (blood pressure exceeding 180 mmHg systolic or 110 mmHg diastolic) were excluded. In addition, anyone with stage IV or V chronic kidney disease (GFR <30 mL/min), liver disease, active cancer, or using immunosuppressant medications was excluded. Furthermore, excessive alcohol consumption (more than 14 drinks per week for men or 7 drinks per week for women), current use of hormone replacement therapy, or uncontrolled thyroid dysfunction (indicated by abnormal thyroid-stimulating hormone levels within 3 mo of enrollment) disqualified potential participants. Individuals with difficulty swallowing capsules, allergies to any supplement ingredients (including glucosamine extract, fucoidan extract, olive extract, artichoke extract, red and white grapes extract, melon concentrate, and hyaluronic acid), or women who were pregnant or planning to become pregnant were also excluded.

#### Study procedures.

**Figure 1.**
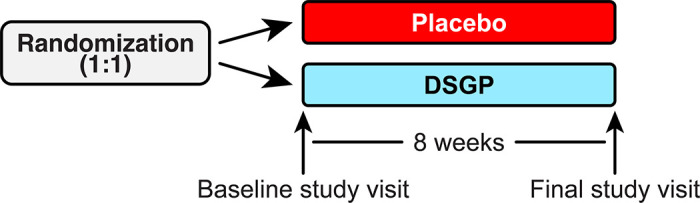
Experimental design of the clinical trial. Following randomization to either dietary supplementation of glycocalyx precursors (DSGPs) treatment or placebo, and at least 24 h prior, but within 7 days of their baseline and final study visits, participants were fitted with a 24-h ambulatory blood pressure monitor for blood pressure assessments.

Participants were randomized 1:1 to either DSGP treatment or placebo (Fig. 1). Preparation and administration of the supplement/placebo capsules were directed by the VA Hospital Investigational Pharmacy. All members of the investigative team involved in data acquisition and analysis were blinded to group assignment, and participants were not informed of their group status. Following randomization and after baseline study visits, participants were provided DSGP in the form of Endocalyx (Microvascular Health Solutions, Alpine, UT) or a matching placebo (Nu-Mag, 10 mg; rice flour, 830 mg; and magnesium stearate veggie powder, 10 mg). Participants were instructed to take three capsules of DSGP twice daily (3,712.5 mg/day), preferably with breakfast and dinner, or placebo for 8 wk. One DSGP capsule contained fucoidan extracted from *Laminaria japonica* (106.25 mg), glucosamine sulfate (375.0 mg), hyaluronic acid (17.5 mg), and a blend of superoxide dismutase and polyphenols (120.0 mg). Subjects were instructed to return any unconsumed capsules at the final study visit. Adherence was determined by capsule count, wherein the total number of capsules ingested was calculated as the number of capsules provided minus the number of capsules returned. Percent adherence was determined as the ratio of capsules ingested to the total capsules prescribed to be ingested × 100. The DSGP dosage for this study was determined through the conversion of preclinical results in diabetic mice, which received 100 mg/kg/day of DSGP for 4 wk. Following 8 wk of supplementation, participants returned to the laboratory for final study visit measurements. In preparation for the study visits, participants were asked to fast overnight (at least 8 h), abstain from tobacco and caffeine intake the morning of the visit, avoid exercise and alcohol consumption for at least 24 h prior, and withhold their diabetes medications the morning of their visit. Participants were also instructed to maintain their usual diet and physical activity levels during the intervention.

##### Twenty four hour ambulatory blood pressure monitoring.

At least 24 h prior, and within 7 days of their baseline and final study visits, participants were fitted with a 24-h ambulatory blood pressure monitor (ABPM 7100, Baxter International, Deerfield, IL) cuff applied to the dominant upper arm by a trained technician. The circumference of the upper arm was measured via a tape measure to ensure the appropriate cuff size was fitted. The ABPM devices were programmed to follow a specific schedule for blood pressure readings over a 24-h period. The devices were set to take a reading every 30 min during waking hours and every 60 min during sleeping hours. In case a reading failed on the first try, the devices were designed to automatically reattempt the measurement within 2 min. Participants were instructed to maintain their standard lifestyle patterns. Participants returned the ABPM device at the time of the baseline, and final visits (Fig. 1), and data were downloaded and analyzed with technical software (CardioPerfect WorkStation) (27). At the baseline assessment, the average number of blood pressure measurements obtained was 28.4 ± 1.2 during the day and 8.3 ± 1.1 during the sleeping hours. At the time of the final assessment, the average number of measurements obtained was 28.9 ± 1.1 during the day and 7.7 ± 0.5 during the sleeping hours. The nocturnal systolic blood pressure dipping rate was calculated as follows: 100 × (daytime − nighttime systolic blood pressure)/(daytime systolic blood pressure).

#### Assessment of carotid-to-femoral pulse wave velocity and FMD.

Carotid-to-femoral pulse wave velocity (cfPWV) was measured using a SphygmoCor XCEL system (AtCor Medical, Itasca, IL) to assess aortic stiffness, as described previously (28). For the assessment of brachial artery FMD, a two-dimensional/Doppler ultrasound (GE Logiq P5) was used, following prior methods and published guidelines (28, 29). Briefly, an 11-MHz linear array transducer was secured with a clamp and placed over the brachial artery. A rapid inflating cuff (Hokanson) was placed on the forearm ∼5 cm distal to the antecubital fossa. In duplex mode, using a pulsed frequency of 5 MHz and corrected for a 60° insonation angle, simultaneous diameter and velocity signals were obtained. The sample volume was adjusted to encompass the entire vessel lumen without extending beyond the walls, and the cursor was set at mid-vessel. Real-time capture software (Elgato Video Capture, Elgato, CA) was used to record 2 min of ultrasound imaging and then the cuff was inflated to a pressure of 250 mmHg for 5 min. Three minutes of ultrasound imaging were collected following cuff deflation. Next, as previously described (30), patients were prepared for superficial femoral artery FMD assessment. Specialized edge-detection software (Cardiovascular Suites 4, Quipu srl, Pisa, Italy) was used for offline analysis of recorded vascular variables. FMD percent change was calculated as follows:

$$\text{FMD }(\%)=100\times \frac{D_{\text{peak}}-D_{\text{bl}}}{D_{\text{bl}}}$$

where *D*_peak_ and *D*_bl_ are the peak and baseline diameters reported in mm. The shear rate was estimated as:

$$\gamma =\frac{4\upsilon }{D}$$

where γ is the shear rate reported in s^−1^, *υ* is mean blood velocity in cm/s, and *D* is the diameter in cm.

#### In vivo glycocalyx thickness.

The integrity of the endothelial glycocalyx was assessed via bedside intravital microscopy using a side-stream dark field camera (CapiScope HVCS, KK Technology, Honiton, UK) to visualize the sublingual microvasculature, as previously described (31). Briefly, side-stream dark field video-microscopy detects hemoglobin found in moving red blood cells (RBC) within the sublingual microcirculation via green light emitting stroboscopic diodes at a wavelength of 540 nm. Image acquisition and analysis are automated using a GlycoCheck system (MicroVascular Health Solutions, Alpine, UT) based on predefined criteria for motion, intensity, and focus. Following analysis, the thickness of the endothelial glycocalyx in microvessels with internal diameters ranging from 4 to 25 μm is reported as perfused boundary region (PBR). PBR represents the distance between flowing RBC and the physical width of the negatively charged glycan structures (glycosaminoglycans and sialic acid) that conform the glycocalyx. A larger PBR value indicates deeper penetration of RBC into the glycocalyx, signifying its degradation and thinning (32). Penetration of RBC into the glycocalyx can be velocity-dependent, thus, to minimize possible flow-dependent variability in PBR estimation, PBR_dynamic_ was used (33). Microvascular vessel density was obtained from the number of measurements sites, with each microvessel measurement site representing 10 μm of length (31). The cumulative microvessel length was divided by the total area recorded to calculate microvascular vessel density. The absolute value for measuring capillary blood volume (CBV_abs_) was determined from the number of capillary segments multiplied by the capillary segment length and the segment-specific capillary cross-sectional area. This value was then multiplied with the CBV ratio that accounts for the average RBC velocity (*V*_RBC_) in larger blood vessels versus small capillaries [=*V*_RBC_ (*D* ≥ 10 µm)/*V*_RBC_(*D* ≤ 7 µm)]. An increase in CBV relative to larger vessel blood volume increases the CBV ratio. Multiplying CBV_abs_ with the CBV ratio gives a static CBV (CBV_static_) value. To take the recruitment capabilities of additional capillaries into account, capillary recruitment (CR) was estimated by measuring the slope of the relationship between *V*_RBC_ (*D* ≤ 7 µm) and *V*_RBC_ (*D* ≥ 10 µm). Multiplying CBV_static_ by (1 + CR) calculates dynamic CBV (CBV_dynamic_) (33). Finally, CBV and PBR proportions can change inversely, thus, the quotient of CBV/PBR was used to calculate one overall dynamic microvascular health score (MVHS_dynamic_) (33).

#### Assessment of leg blood flow, total peripheral resistance, and skeletal muscle microvascular perfusion in response to insulin.

Insulin-stimulated leg blood flow was assessed as another indicator of vascular function as described previously (28). Briefly, intravenous catheters were inserted in both antecubital veins for blood sampling and infusions of insulin and dextrose. Approximately 20 min after catheter placement, assessments of blood flow in the superficial femoral artery (4-min recording period) followed by microvascular perfusion in the quadriceps muscle (vastus lateralis) were obtained via a two-dimensional/Doppler ultrasound and contrast-enhanced ultrasound, respectively (28). Then, insulin (Novolin-R U-100) was prepared via dilution in 250 mL of 0.9% saline along with 5 mL of whole blood obtained from the subject to a final concentration of 500 mU/mL. Insulin was then infused at a constant dosage of 80 mU/m^2^ body surface area/min. Following the 60-min infusion, superficial femoral artery blood flow and quadriceps muscle microvascular perfusion were reassessed. Blood flow (reported in mL/min) was calculated as 3.14 × [diameter (cm)/2]^2^ × mean blood velocity (cm/s) × 60. Beat-to-beat arterial pressure waveforms were obtained noninvasively using finger photoplethysmography (Human NIBP, ADInstruments) and calibrated to mean arterial pressure (MAP), calculated as an average of upper arm sphygmomanometer blood pressures [(1/3 × systolic blood pressure) + (2/3 × diastolic blood pressure)]. Leg vascular conductance was calculated as leg blood flow/MAP across the recording period. Total peripheral resistance (TPR) was estimated using the Modelflow method (LabChart, ADInstruments), which integrates sex and age. Whole blood glucose was measured every 5 min (YSI Inc., Yellow Springs, OH) and maintained at fasted levels. This was achieved by variable infusion rates of a 20% dextrose solution. Plasma was obtained and stored at −80°C for later analysis.

#### Measurement of fasting plasma nitrite and biochemical parameters.

Plasma nitrite concentrations, a surrogate of NO, were assessed using an ozone-based reductive chemiluminescence NO analyzer (CLD88, Eco Physics) according to the manufacturer guidelines and previously described (34, 35). Plasma samples (100 µL) were injected in triplicate into a purge vessel containing 3.5 mL of glacial acetic acid and 0.5 mL ascorbic acid (0.5 mM) (36, 37), which was then purged with pure nitrogen in-line with the CLD88 gas-phase NO analyzer. The chemiluminescence signal was acquired using eDAQ ChartTM v5.5.27 software, and NO quantification was performed using the flow injection analysis (FIA) software extension (ADInstruments, Australia). The FIA software calculated the area under the curve for each sample peak, which was then converted to a concentration using a calibrated standard curve generated with known sodium nitrite standards. Plasma samples obtained during the insulin infusion were assessed for insulin concentrations using a commercially available kit (ALPCO Cat. No. 80-INSHU-E10.1, Salem, NH). Concentrations of endothelin-1 (No. EIAET1, Invitrogen, Thermo Fisher Scientific), Glucose (No. 10009582, Glucose Colorimetric Assay, Caymen Chemical), glypican-1 (No. ab270217, SimpleStep, Abcam), and hyaluronan (No. DHYAL0, Quantikine, Bio-Techne) in plasma were assessed using commercially available ELISA kits following manufacturer instructions.

### Statistical Analysis

All power analyses used an α of 0.05 and 80% power for the clinical trial. We determined that a sample size of 10 subjects per group would provide 82% power and be sufficient to detect significant differences in outcomes based on preclinical data and preliminary unpublished data in humans. We enrolled 24 subjects to account for any potential dropouts. Data are presented as means ± standard error of the mean (SE). Shapiro–Wilk test was performed for the assessment of data distribution. Treatment-related differences in outcomes were determined using two-tailed paired and unpaired Student’s *t* test and one-way ANOVA or two-way ANOVA with repeated-measures, as appropriate. Bonferroni post hoc tests were performed when significant interactions were found. When data were not normally distributed, nonparametric tests, Mann–Whitney *U* (Wilcoxon rank-sum test), and Wilcoxon signed-rank tests were performed. The results were considered significant when *P* < 0.05. Statistical analyses were performed using GraphPad Prism (v.10.0) while the investigative team remained blinded to treatment groups.

## RESULTS

**Figure 2.**
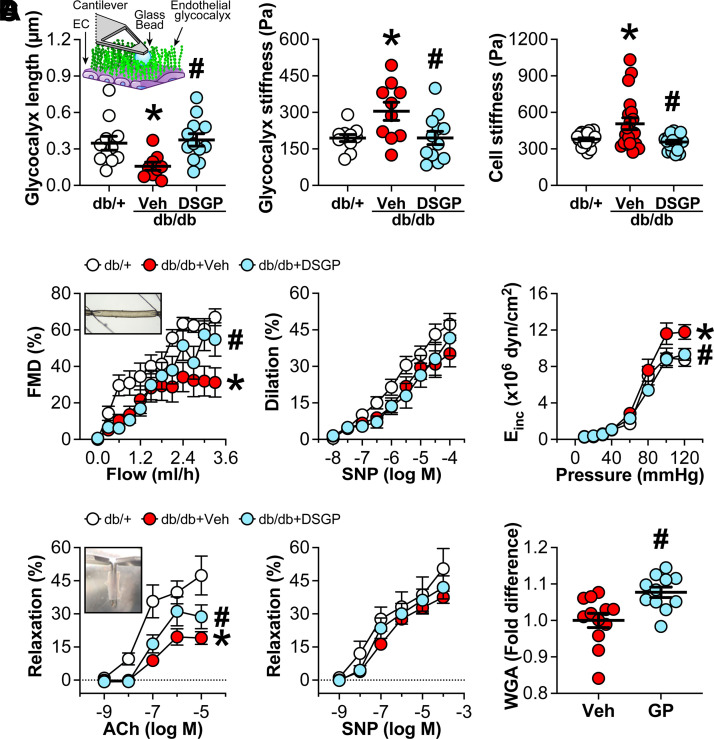
Vascular effects of treatment with dietary supplementation of glycocalyx precursors (DSGPs) vs. vehicle (Veh) in db/db mice, and the effect of glycocalyx precursors on glycocalyx coverage in cultured endothelial cells. *A*: endothelial glycocalyx length, as well as glycocalyx and cell cortical stiffness, was assessed in en face aortic explants (schematic representation shown in the *inset*) using atomic force microscopy in db/+ control mice and in db/db mice treated with DSGP vs. vehicle for 4 wk. Statistical analysis was performed using one-way ANOVA with Bonferroni post hoc test; *n* = 9–20/group. *B*: flow-mediated dilation (FMD) and sodium nitroprusside (SNP)-induced dilation in isolated mesenteric arteries. Data are expressed as percentages of dilation from phenylephrine preconstriction in response to increasing flow rates or SNP concentrations. Mesenteric artery stiffness was assessed via the incremental modulus of elasticity (*E*_inc_) at increasing intraluminal pressures under calcium-free conditions. Statistical analysis was performed using two-way ANOVA with repeated measures and Bonferroni post hoc test; *n* = 8–9/group. *C*: aortic relaxation was assessed in response to increasing concentrations of acetylcholine (ACh) and SNP following preconstriction with U46619. Statistical analysis was performed using two-way ANOVA with repeated measures and Bonferroni post hoc test; *n* = 6–16/group. *D*: human umbilical vein endothelial cells exposed to neuraminidase to induce glycocalyx degradation were treated with vehicle vs. the cocktail of glycocalyx precursors (GP) for 48 h. Glycocalyx coverage was determined by staining with fluorescent wheat germ agglutinin (WGA) and fluorescence intensity quantification. Statistical analysis was performed using unpaired Student’s *t* tests; *n* = 11–12/condition. Data are presented as means ± SE. Individual data points are also presented as appropriate. *P < 0.05 vs. db/+, #*P* < 0.05 vs. db/db + Veh or Veh.

As expected, body weight at the time of euthanasia was higher in the db/db mice (51.10 ± 0.87 g) compared with the db/+ mice (24.97 ± 0.47 g). No effect of DSGP was observed on this parameter (db/db + DSGP: 50.30 ± 1.03 g). Also as expected, plasma glucose at the time of euthanasia was higher in the db/db mice (658.6 ± 38.9 mg/dL) compared with db/+ mice (243.0 ± 6.5 mg/dL), whereas mice treated with DSGP displayed even greater glucose levels (737.5 ± 25.3 mg/dL; *P* < 0.05). As depicted in Fig. 2*A*, vehicle-treated db/db mice exhibited a reduced endothelial glycocalyx length compared with db/+ control mice (*P* < 0.05), as assessed via AFM in en face aortic explants. This deficit was restored in db/db mice treated with DSGP for 4 wk (*P* < 0.05). Glycocalyx stiffness and cell cortical stiffness were also higher in vehicle-treated db/db mice, relative to db/+ controls (*P* < 0.05), and again normalized by DSGP treatment (*P* < 0.05). As shown in Fig. 2*B*, FMD in isolated mesenteric arteries was reduced in vehicle-treated db/db mice compared with db/+ controls (*P* < 0.05), indicative of impaired shear stress mechanotransduction. This impairment was corrected with DSGP treatment (*P* < 0.05). No group differences were noted in vasodilatory responses to SNP (*P* > 0.05). The incremental modulus of elasticity, a measurement of structural stiffness, was elevated in vehicle-treated (*P* < 0.05) but not DSGP-treated db/db mice (*P* > 0.05). As displayed in Fig. 2*C*, vehicle-treated db/db mice exhibited impaired ACh-induced relaxation in aortic rings, relative to db/+ controls (*P* < 0.05). This impairment was partially corrected with DSGP treatment (*P* < 0.05). No group differences were noted in relaxation responses to SNP (*P* > 0.05). Finally, as shown in Fig. 2*D*, cultured endothelial cells treated with the cocktail of glycocalyx precursors for 48 h displayed greater glycocalyx coverage, as assessed using WGA staining, compared with vehicle-treated cells (*P* < 0.05).

**Figure 3.**
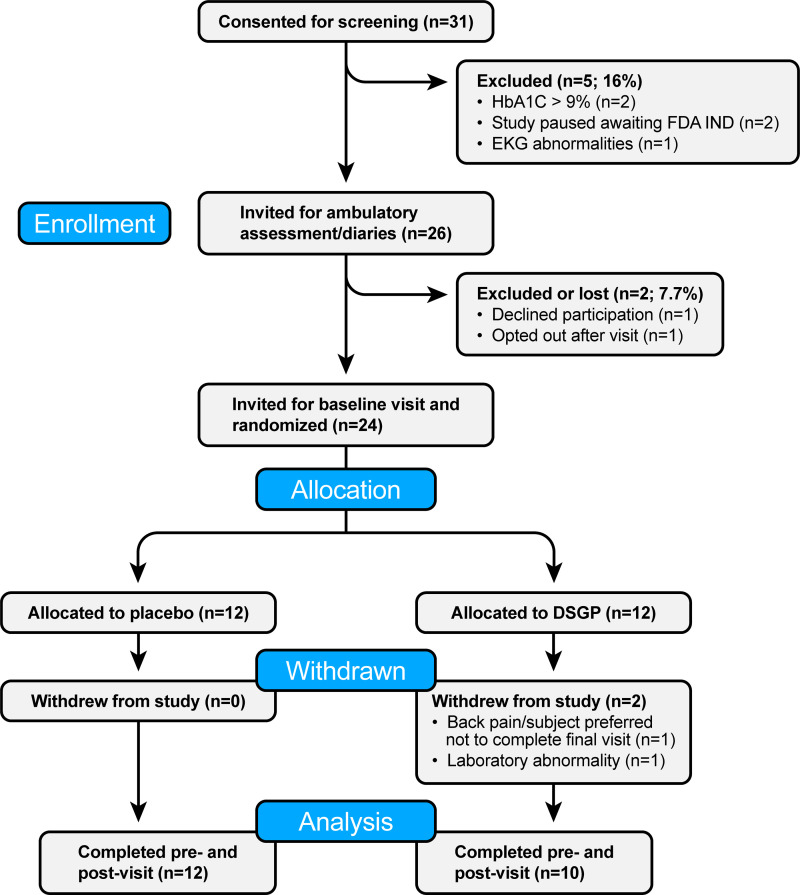
Flow diagram for the clinical trial. DSGP, dietary supplementation of glycocalyx precursors.

**Figure 4.**
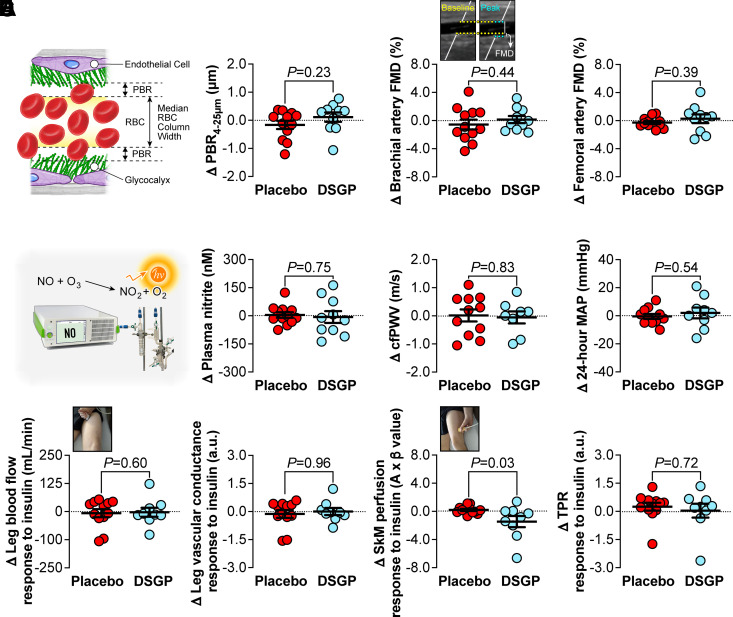
Vascular effects of dietary supplementation of glycocalyx precursors (DSGPs) treatment vs. placebo in Veterans with type 2 diabetes. *A*: delta change in perfused boundary region (PBR) from pre- to postintervention. Statistical analysis was performed using unpaired Student’s *t* tests; *n* = 10–12/group. *B*: delta change in brachial and femoral artery flow-mediated dilation (FMD) from pre- to postintervention. Statistical analysis was performed using unpaired Student’s *t* tests; *n* = 10–12/group. *C*: delta change in plasma nitrite from pre- to postintervention. Statistical analysis was performed using unpaired Student’s *t* tests; *n* = 10–12/group. *D*: delta change in carotid-to-femoral pulse wave velocity (cfPWV) from pre- to postintervention. Statistical analysis was performed using unpaired Student’s *t* tests; *n* = 8–11/group. *E*: delta change in 24-h average mean arterial blood pressure (MAP) from pre- to postintervention. Statistical analysis was performed using unpaired Student’s *t* tests; *n* = 9–11/group. *F*: delta change in leg blood flow and vascular conductance, skeletal muscle (SkM) perfusion, and total peripheral resistance (TPR) response to insulin from pre- to postintervention. Measurements were performed at baseline and at 60 min of systemic insulin infusion (with the coinfusion of dextrose to maintain euglycemia). The difference between time points was then calculated to capture the insulin response as the outcome variable. Statistical analysis of normally distributed data (SkM perfusion) was performed using unpaired Student’s *t* tests. Non normally distributed data (leg blood flow, vascular conductance, and TPR) were analyzed using unpaired Mann–Whitney *U* (Wilcoxon rank-sum) test; *n* = 9–12/group. Data are presented as means ± SE. Individual data points are also presented. *P* values for all comparisons are shown.

A total of 24 Veterans with T2D were randomized to either DSGP treatment or placebo. Two subjects allocated to DSGP withdrew from the study, leaving 10 and 12 subjects for analysis (Fig. 3). The reasons for exclusion are summarized in Fig. 3. Table 1 summarizes subject characteristics, anthropometrics, and blood profile parameters before and after DSGP treatment versus placebo for 8 wk. A significant interaction effect was observed for both homeostatic model assessment for insulin resistance (HOMA-IR) and fasting insulin (*P* < 0.05); however, the Bonferroni post hoc test did not reveal any significant differences for either outcome (*P* > 0.05). DSGP did not have a significant impact on the other parameters assessed (*P* > 0.05). In contrast to the findings in mice, DSGP did not have an effect on vascular outcomes in this cohort of subjects with T2D (*P* > 0.05). Indeed, as depicted in Fig. 4*A*, the delta change in PBR from pre- to postintervention was not different between placebo and DSGP-treated subjects (*P* > 0.05). Similarly, relative to placebo, DSGP treatment did not affect brachial nor femoral artery FMD (*P* > 0.05, Fig. 4*B*), plasma nitrite (*P* > 0.05, Fig. 4*C*), cfPWV (*P* > 0.05, Fig. 4*D*), or 24-h average MAP (*P* > 0.05, Fig. 4*E*). We also assessed leg blood flow, leg vascular conductance, skeletal muscle perfusion, and total peripheral resistance in response to systemic insulin infusion, and no improvements were observed after DSGP treatment (*P* > 0.05, Fig. 4*F*). Table 2 further depicts cardiovascular and hemodynamic outcomes.

**Table 1.** Subject characteristics, anthropometrics, and blood profile parameters before and after DSGPs vs. placebo treatment in Veterans with type 2 diabetes

	PlaceboMeans ± SE (*n* = 12)		DSGPMeans ± SE (*n* = 10)	
	Before	After	Before	After
Race (*n*, %)Caucasian/Non-Hispanic	(12, 100%)		(10, 100%)	
Age, yr	61 ± 2		66 ± 2	
Length of T2D diagnosis, yr	13 ± 2		15 ± 3	
Capsule consumption adherence, %		97.3 ± 0.88		97.9 ± 0.83
Height, cm	175.75 ± 2.42		174.70 ± 1.84	
Body weight, kg	107.35 ± 4.17	106.80 ± 4.30	103.06 ± 5.99	103.71 ± 5.61
Body mass index, kg/m^2^	34.54 ± 1.24	34.42 ± 1.22	33.52 ± 1.64	33.75 ± 1.56
Body fat mass, %	39.81 ± 1.89	39.35 ± 1.77	37.71 ± 2.03	38.72 ± 2.02*
Lean body mass, kg/m^2^	19.69 ± 0.51	19.94 ± 0.44	20.03 ± 0.79	20.16 ± 0.75
Systolic BP, mmHg	130 ± 3	129 ± 5	143 ± 6	144 ± 8
Diastolic BP, mmHg	78 ± 2	78 ± 2	80 ± 2	82 ± 2
Heart rate, beats/min	67 ± 2	66 ± 3	59 ± 2	61 ± 2
Fasted blood glucose, mg/dL	134.09 ± 9.15	131.00 ± 8.39	126.98 ± 15.89	134.74 ± 14.43
Fasted insulin, μU/mL	12.37 ± 1.88	9.40 ± 1.49	12.25 ± 3.51	14.68 ± 4.30
HOMA-IR, mg/dL	3.99 ± 0.57	3.04 ± 0.53	3.67 ± 1.07	4.62 ± 1.22
Hemoglobin A1C, %	7.48 ± 0.21		7.01 ± 0.26	
Estimated glomerular filtration rate, mL/min	85.42 ± 5.66		77.70 ± 8.24	
Glypican-1, ng/mL	14.49 ± 0.88	14.17 ± 0.85	15.88 ± 1.19	15.48 ± 0.75
Hyaluronan, ng/mL	41.17 ± 8.55	40.27 ± 5.86	47.48 ± 8.65	46.74 ± 8.77
Endothelin-1, pg/mL	63.63 ± 5.05	65.64 ± 4.41	65.13 ± 5.65	63.44 ± 8.05
Nitrite, nM	61.42 ± 12.56	65.58 ± 15.26	95.40 ± 17.66	89.00 ± 26.57
Medications			
Biguanide	10		7	
Glucagon-like peptide-1 receptor agonist	9		5	
Sodium-glucose co-transporter 2 inhibitor	10		1	
Sulfonylurea	2		1	
Insulin	6		6	
Angiotensin-converting-enzyme inhibitor	6		4	
Beta blocker	5		5	
Angiotensin II receptor antagonist	2		1	
Calcium channel blocker	1		5	
Thiazide	2		7	
Statins	11		9	

Data are presented as means ± SE. BP, blood pressure; HOMA-IR, homeostatic model assessment for insulin resistance; T2D, type 2 diabetes.

* *P* < 0.05 vs. before dietary supplementation of glycocalyx precursors (DSGPs).

**Table 2.** Cardiovascular and hemodynamic outcomes before and after DSGP vs. placebo treatment in Veterans with type 2 diabetes

	PlaceboMeans ± SE (*n* = 12)		DSGPMeans ± SE (*n* = 10)	
	Before	After	Before	After
Femoral Artery				
Baseline diameter, mm	5.99 ± 0.26	5.95 ± 0.25	6.65 ± 0.22	6.63 ± 0.22
Peak diameter, mm	6.12 ± 0.26	6.05 ± 0.24	6.85 ± 0.24#	6.85 ± 0.25#
Absolute change in diameter, mm	0.13 ± 0.02	0.11 ± 0.02	0.20 ± 0.03#	0.22 ± 0.04#
FMD, %	2.14 ± 0.27	1.87 ± 0.34	2.93 ± 0.39#	3.20 ± 0.47#
Mean shear, s^−1^	44.19 ± 8.02	42.57 ± 5.25	51.51 ± 11.57	39.73 ± 6.51
Shear AUC, ×10^3^ au	10.43 ± 2.6	9.00 ± 1.74	12.03 ± 4.42	7.07 ± 1.71
Time-to-peak dilation, s	98.42 ± 10.41	106.79 ± 12.62	100.40 ± 11.5	89.85 ± 13.22
Brachial artery				
Baseline diameter, mm	4.25 ± 0.16	4.24 ± 0.2	4.70 ± 0.23	4.62 ± 0.21
Peak diameter, mm	4.38 ± 0.16	4.35 ± 0.2	4.84 ± 0.22	4.77 ± 0.21
Absolute change in diameter, mm	0.13 ± 0.01	0.11 ± 0.02	0.15 ± 0.03	0.15 ± 0.03
FMD, %	3.20 ± 0.38	2.63 ± 0.54	3.26 ± 0.76	3.40 ± 0.72
Mean shear, s^−1^	140.32 ± 19.71	128.18 ± 16.39	119.36 ± 12.34	115.93 ± 10.74
Shear AUC, ×10^3^ au	25.21 ± 2.98	19.83 ± 2.37	22.22 ± 1.83	19.82 ± 2.74
Time-to-peak dilation, s	83.13 ± 8.68	77.33 ± 10.08	92.90 ± 8.21	88.75 ± 9.77
GlycoCheck-derived variables				
CBV_dynamic_, 10^3^ μm^3^/mm^2^	15.50 ± 1.87	15.55 ± 3.03	18.33 ± 2.6	16.50 ± 1.77
MVHS_dynamic_, µm	2.82 ± 0.39	2.97 ± 0.57	3.50 ± 0.47	3.06 ± 0.4
Capillary density 4–25 µm, µm/mm^2^	190.24 ± 36.97	170.62 ± 42.05	215.16 ± 39.53	174.67 ± 26.66
PBR_4–25 µm_, µm	2.31 ± 0.15	2.15 ± 0.14	2.08 ± 0.11	2.19 ± 0.09
PBR_4–7µm_, µm	1.18 ± 0.09	1.06 ± 0.07	1.14 ± 0.09	1.05 ± 0.08
PBR_10–25 µm_, µm	2.74 ± 0.11	2.62 ± 0.12	2.74 ± 0.10	2.70 ± 0.11
Additional cardiovascular outcomes				
cfPWV, m/s	8.46 ± 0.33	8.49 ± 0.46	8.79 ± 0.58	8.71 ± 0.46
24-h MAP, mmHg	96 ± 3	96 ± 4	101 ± 3	104 ± 4
Daytime SBP, mmHg	130 ± 4	131 ± 6	144 ± 5	144 ± 7
Nighttime SBP, mmHg	126 ± 3	126 ± 4	139 ± 8	145 ± 7
Daytime DBP, mmHg	80 ± 3	79 ± 3	82 ± 3	84 ± 3
Nighttime DBP, mmHg	76 ± 3	75 ± 3	78 ± 4	84 ± 4
Day-night SBP dip, %	3.1 ± 1.6	3.2 ± 2.4	4.4 ± 2.9	−1.2 ± 4.5
Leg blood flow response to insulin, mL/min	16.93 ± 15.38	9.55 ± 9.03	5.98 ± 11.92	2.97 ± 12.14
Leg vascular conductance response to insulin, au	0.20 ± 0.18	0.07 ± 0.09	0.01 ± 0.12	0.02 ± 0.11
SkM perfusion response to insulin, A × β value	−0.17 ± 0.19	0.04 ± 0.18	1.58 ± 0.72†	0.12 ± 0.26*
TPR response to insulin, au	0.02 ± 0.11	0.26 ± 0.13	0.19 ± 0.31	0.23 ± 0.09

Data are presented as means ± SE. Measurements were performed at baseline and at 60 min of systemic insulin infusion (with the coinfusion of dextrose to maintain euglycemia). The difference between time points was then calculated to capture the insulin response as the outcome variable. AUC, area under the curve; CBV, capillary blood volume; cfPWV, carotid-femoral pulse wave velocity; DBP, diastolic blood pressure; DSGPs, dietary supplementation of glycocalyx precursors; FMD, flow-mediated dilation; MAP, mean arterial pressure; MVHS, microvascular health score; PBR, perfused boundary region; SBP, systolic blood pressure; SkM, skeletal muscle; TPR, total peripheral resistance.

* *P* < 0.05 vs. before DSGP; †*P* < 0.05 vs. before placebo; #*P* < 0.05, main effect of group.

## DISCUSSION

The primary findings of this investigation are that DSGP increases endothelial glycocalyx length, improves FMD, and reduces arterial stiffness in db/db mice, a rodent model of T2D. However, these observations are not translatable to individuals with T2D. Indeed, 8 wk of DSGP did not enhance the endothelial glycocalyx, nor improve any indices of vascular function in Veterans with T2D.

The impetus for this work stems from the increasing evidence that degradation of the endothelial glycocalyx, a phenomenon well documented in T2D (12, 16–21, 26, 38), contributes to endothelial dysfunction and, consequently, to the development of CVD (39). One of the key functions of the endothelial glycocalyx is to serve as a mechanosensor of shear stress (13). A plethora of studies, both in vitro and in vivo, demonstrate shear stress is a crucial mechanical signal to the vascular endothelium that promotes the production of nitric oxide and other vascular protective molecules (40, 41). As such, impaired shear stress mechanotransduction, caused in part by the destruction of glycocalyx structures, is considered an important contributor to endothelial dysfunction and cardiovascular complications. This positions the endothelial glycocalyx as an attractive vascular component for intervention. Certainly, therapeutic strategies designed to restore the endothelial glycocalyx could have a substantial impact on the prevention and treatment of T2D-associated CVD.

After surveying available strategies to modify the glycocalyx length with translational potential, we first directed our efforts toward targeting neuraminidase. This enzyme cleaves sialic acid from glycocalyx structures, and it is increased in the circulation of individuals with T2D (26, 42). Recently, we corroborated in cultured endothelial cells that neuraminidase exposure diminishes glycocalyx coverage and that, in isolated arteries, this is accompanied by impaired FMD, indicative of impaired shear stress mechanotransduction (26). Subsequently, we showed that inhalation of the FDA-approved neuraminidase inhibitor, Zanamivir, reduced plasma neuraminidase activity, enhanced endothelial glycocalyx length, and improved FMD in db/db mice (26). However, our attempt to translate these findings into humans was not successful. Zanamivir inhalation at the currently FDA-recommended dose did not reduce plasma neuraminidase activity nor improve glycocalyx length or FMD in individuals with T2D, likely due to the limited concentration of plasma Zanamivir achieved by such dosing (26). These negative findings in humans led us to consider DSGP as another potential strategy to restore the endothelial glycocalyx in T2D.

Our focus on DSGP was founded partly on the preclinical work from Machin et al. (43), demonstrating that feeding aged mice with a cocktail of glycocalyx precursors available as a dietary supplement enhanced glycocalyx thickness and improved vascular function. As an early step, we used our endothelial cell culture model of glycocalyx degradation, which consists of endothelial exposure to neuraminidase (26). We showed that treatment of glycocalyx-degraded endothelial cells with the cocktail of glycocalyx precursors, which contains glucosamine sulfate, fucoidan, superoxide dismutase, and high-molecular weight hyaluronan, promoted glycocalyx growth. Next, we showed that treatment of db/db mice with DSGP for 4 wk, similar to findings in aged mice (43), augmented endothelial glycocalyx thickness (i.e., length) as assessed via AFM in aortic explants. The increase in glycocalyx length with DSGP was associated with reduced glycocalyx and cellular stiffness. This finding is of interest as data are available suggesting that a stiff glycocalyx may compromise its ability to transmit mechanical forces into the cell (12, 44). Along these lines, we also showed that DSGP improved FMD in isolated mesenteric arteries without influencing endothelium-independent dilation to SNP. We also report that the improvement in endothelial function was accompanied by a reduction in arterial stiffness. Notably, all these beneficial vascular effects of DSGP in diabetic mice occurred even despite a further elevation of blood glucose levels.

The overall positive results in mice led us to take the next logical step, which is to assess the efficacy of DSGP in patients with T2D using a randomized controlled trial. In contrast to the hypothesis, DSGP treatment for 8 wk did not enhance the integrity of the endothelial glycocalyx, as determined by the PBR assessed in the sublingual circulation using the GlycoCheck, nor did it improve indices of vascular function in Veterans with T2D. Our pilot study, while small in sample size, involved multiple vascular outcomes, including FMD in the brachial and superficial femoral arteries, insulin-stimulated leg blood flow and skeletal muscle perfusion via Doppler and contrast-enhanced ultrasound, and arterial stiffness as assessed using cfPWV. Despite the comprehensive vascular phenotyping, the results consistently demonstrated a lack of an effect of DSGP. Likewise, the intervention did not beneficially impact body composition, metabolic outcomes, or lipid profiles. Neither resting (Table 1) nor ambulatory blood pressure (Table 2) were affected by DSGP. Of note, and as shown in Table 1, subjects randomly assigned to placebo tended to have lower systolic blood pressure compared with those assigned to DSGP (*P* = 0.08). Although speculative, this may be attributable to the fact that, by chance, more subjects assigned to placebo were using SGLT2 inhibitors and GLP-1 receptor agonists, agents with documented blood pressure-lowering effects (45, 46).

Coincidently, during the preparation of this manuscript, the article by Gimblet et al. (47) was published demonstrating, in a similar size randomized controlled trial (*n* = 23), that 3,712.5 mg/day of DSGP for 12 wk failed to enhance glycocalyx integrity (also based on the measurement of PBR using the GlycoCheck), improve brachial artery FMD, or reduce cfPWV in older adults. We consider the similarity in negative findings between the two simultaneously and independently conducted clinical trials as a strength in that it reinforces the notion that this glycocalyx-targeted therapy is not efficacious for improving human vascular function. Our findings, together with findings by Gimblet et al. (47) and our previous clinical study using the neuraminidase inhibitor Zanamivir (26), show that restoring the endothelial glycocalyx in humans remains an unresolved challenge. However, this is not a universal finding. A recently published manuscript by van der Velden et al. (38) demonstrated in a cohort of South-Asian Surinamese descent individuals with T2D that 2,475 mg/day of DSGP for 12 wk reduced PBR, indicative of increased endothelial glycocalyx integrity. Although we do not have a strong argument for explaining this discrepancy in results, we speculate that the difference could be attributed to the fact that the participants in our clinical trial had a higher usage rate of antidiabetic agents with demonstrated cardiovascular benefits such as sodium-glucose transporter-2 inhibitors and glucagon-like peptide-1 receptor agonists. Other distinctions between the two studies that are worth mentioning and that may have contributed to differing results include the shorter duration of T2D in the population studied by van der Velden et al. (38) and the longer duration of supplementation.

Several aspects of the present investigation warrant further consideration. First, although the clinical trial was open for recruitment to both sexes, the vast majority of participants were men. This was partly because ∼80% of patients at the VA are males, and no specific recruitment strategies were put in place to maintain an equal distribution of men and women, which can be considered a weakness of this pilot study. It is also important to note that the preclinical studies were conducted exclusively in female mice. It is possible that the effect of DSGP could have differed in male db/db mice. However, given the negative results of our DSGP clinical trial in a predominantly male Veteran cohort, it is unlikely that positive results in a male model of diabetes would have any meaningful translational impact. Still, future studies are needed to evaluate whether the impact of T2D on glycocalyx integrity is influenced by sex.

Second, our preclinical studies were performed in a rodent model of T2D characterized by severe insulin resistance and hyperglycemia (i.e., db/db mice). In the current day and age, it is unlikely that individuals with T2D who have access to standard-of-care medical therapies will present with the degree of hyperglycemia exhibited by the preclinical model. Therefore, it is possible that the positive preclinical results we observed may be attributed to the degree of hyperglycemia, glucotoxicity, and glycocalyx destruction.

Third, in the clinical study, the PBR-based assessment of endothelial glycocalyx dimensions was performed in the sublingual circulation following established procedures (48). At this time, we cannot attest that an index of glycocalyx integrity in the sublingual circulation indicates glycocalyx integrity in other vascular beds. Yet, it is reasonable to infer that if the intervention is systemic (as is the case with DSGP), an improvement in glycocalyx integrity in one vascular bed should reflect improvements in glycocalyx integrity in all beds, even if the magnitude of change is variable. In addition, we acknowledge that the use of other methods to assess glycocalyx integrity (15, 49) might have yielded different results.

Finally, Endocalyx, the patented and commercially available dietary supplement used in this study, contains multiple ingredients designed to synthesize, repair, and provide antioxidant support to the endothelial glycocalyx (50). Additional studies could be conducted to study the vascular effects of the different ingredients in isolation using in vitro and/or in vivo models.

In aggregate, the findings presented herein conceptually support the notion that restoration of the endothelial glycocalyx using DSGP can lead to improvements in vascular function in a mouse model of T2D. However, DSGP as a therapeutic strategy to enhance vascular function does not appear to be efficacious in Veterans with T2D, and based on recent data from others, it is likely not efficacious for many other populations. We hope this sum of work stimulates more innovative ideas for therapeutic strategies to effectively boost the endothelial glycocalyx in T2D and other disease states.

## DATA AVAILABILITY

Data will be made available upon reasonable request.

## GRANTS

This work was supported in part by the National Institutes of Health Grant R01HL153264 (to L.A.M.-L. and J.P.). F.I.R.-P. and L.F.-S. were supported by the University of Missouri (MU) Research Excellence Program. N.J.M. was supported by the MU Life Sciences Fellowship Program. The clinical trial portion of this work was supported by the Veterans Affairs Merit Grant 1I01CX002399 (to C.M.-A. and J.P.).

## DISCLOSURES

No conflicts of interest, financial or otherwise, are declared by the authors.

Jaume Padilla is an editor of *Journal of Applied Physiology* and was not involved and did not have access to information regarding the peer-review process or final disposition of this article. An alternate editor oversaw the peer-review and decision-making process for this article.

## AUTHOR CONTRIBUTIONS

L.A.M.-L., J.P., and C.M.-A. conceived and designed research; J.A.S., F.I.R.-P., K.B., J.D.G.-V., M.M.-Q., N.J.M., L.F.-S., N.S., and C.A.F. performed experiments; J.A.S., F.I.R.-P., N.J.M., and L.F.-S. analyzed data; J.A.S., F.I.R.-P., K.B., N.J.M., L.F.-S., C.A.F., L.A.M.-L., J.P., and C.M.-A. interpreted results of experiments; J.A.S., F.I.R.-P., and J.P. prepared figures; J.A.S., J.P., and C.M.-A. drafted manuscript; J.A.S., F.I.R.-P., N.J.M., L.F.-S., C.A.F., L.A.M.-L., J.P., and C.M.-A. edited and revised manuscript; J.A.S., F.I.R.-P., K.B., J.D.G.-V., M.M.-Q., N.J.M., L.F.-S., N.S., C.A.F., L.A.M.-L., J.P., and C.M.-A. approved final version of manuscript.
